# Study on the Mass Spectrometry Fragmentation Patterns for Rapid Screening and Structure Identification of Ketamine Analogues in Illicit Powders

**DOI:** 10.3390/molecules28186510

**Published:** 2023-09-08

**Authors:** Yilei Fan, Jianhong Gao, Xianxin Chen, Hao Wu, Xing Ke, Yu Xu

**Affiliations:** 1 Key Laboratory of Drug Prevention and Control Technology of Zhejiang Province, Department of Criminal Science and Technology, Zhejiang Police College, Hangzhou 310053, China; fanyilei@zjjcxy.cn (Y.F.); kexing@zjjcxy.cn (X.K.); 2 Green Pharmaceutical Collaborative Innovation Center of Yangtze River Delta Region, College of Pharmaceutical Science, Zhejiang University of Technology, Hangzhou 310053, China; 3 Key Laboratory of Drug Monitoring and Control of Zhejiang Province, National Anti-Drug Laboratory Zhejiang Regional Center, Hangzhou 310053, Chinachenxianxin1128@163.com (X.C.); 4 Dian Regional Forensic Science Institute Zhejiang, Hangzhou 310007, China; wuhao3@dazd.cn

**Keywords:** ketamine analogues, fragmentation patterns, GC-QTOF/MS, LC-Q-Orbitrap MS/MS, structure identification

## Abstract

Ketamine analogues have been emerging in recent years and are causing severe health and social problems worldwide. Ketamine analogues use 2-phenyl-2-aminocyclohexanone as the basic structure and achieve physiological reactions similar to or even more robust than the prototype of ketamine by changing the substituents on the benzene ring (R_1_ and R_2_) and amine group (R_N1_). Therefore, the mass spectrometry (MS) fragmentation pathways and fragments of ketamine analogues have certain regularity. Eight ketamine analogues are systematically investigated by GC-QTOF/MS and LC-Q-Orbitrap MS/MS with the positive mode of electrospray ionization. The MS fragmentation patterns of ketamine analogues are summarized according to high-resolution MS data. The α-cleavage of carbon bond C_1_-C_2_ in the cyclohexanone moiety and further losses of CO, methyl radical, ethyl radical and propyl radical are the characteristic fragmentation pathways of ketamine analogues in EI-MS mode. The loss of H_2_O or the sequential loss of R_N1_NH_2_, CO and C_4_H_6_ are the distinctive fragmentation pathways of ketamine analogues in ESI-MS/MS mode. Moreover, these MS fragmentation patterns are first introduced for the rapid screening of ketamine analogues in suspicious powder. Furthermore, the structure of the ketamine analogue in suspicious powder is 2-(Methylamino)-2-(o-tolyl)cyclohexan-1-one, which is further confirmed by NMR. This study contributes to the identification of the chemical structure of ketamine analogues, which can be used for the rapid screening of ketamine analogues in seized chemicals.

## 1. Introduction

**Scheme 1:**
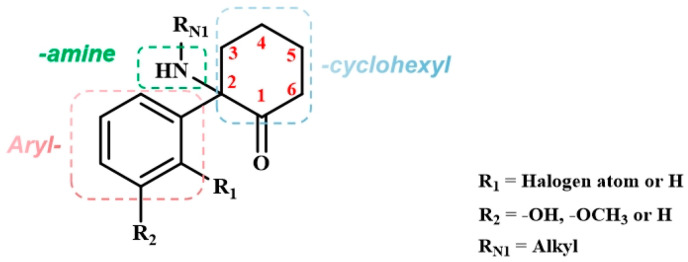
The chemical structure of common ketamine analogues.

In recent years, new psychoactive substances (NPS) are continuing to emerge in the form of stand-alone compounds or mixtures and are gradually replacing traditional drugs as the dominant abuse drug in many countries [1]. Until 2021, the United Nations Office on Drugs and Crime (UNODC) has received reports from 133 countries and regions worldwide, documenting 1079 kinds of new psychoactive substances. Among these, ketamine is one of the most abused new psychoactive substances, reported by 93 countries and regions [2]. Ketamine is a representative substance of arylcyclohexanoneamine derivatives, which acts as an antagonist to the N-methyl-D-aspartate (NMDA) receptor in the human body [3]. Additionally, because of its effects on the central nervous system, which can cause hallucinations, dissociation, and euphoria, ketamine is one of the most widely abused hallucinogens [4,5]. With the increasing attention being paid to the abuse and addiction of ketamine by governments, ketamine is now classified as a strictly controlled substance in many countries worldwide. Recently, the abuse of a class of substances similar in structure and action to ketamine has become apparent [6]. The chemical structure of common ketamine analogues is exhibited in Scheme 1. In order to retain the antagonistic activity against the NAMD receptor, illegal elements mainly modify the substituents on the benzene ring and amino group of ketamine to obtain analogues [7,8]. In the last decade, ketamine analogues such as deschloroketamine (DCK), 2-fluoro-deschloroketamine (2F-DCk), etc., have continued to appear and are utilized for recreation purposes [9]. Moreover, these ketamine analogues all exhibit antagonistic effects on NMDA and achieve the same effects when they are abused recreationally [10].

With the rapid evolution of the chemical structure of ketamine analogues, the quick detection and structure identification of these substances is of great significance. Fourier transform infrared spectroscopy (FT-IR), Raman spectrometer and nuclear magnetic resonance spectrometer (NMR) are powerful tools for the structure identification of unknown substances. Xu et al. (2021) analyzed 28 kinds of fentanyl-class substances by FT-IR and Raman spectrometer, which is the first choice for the rapid identification of fentanyl-class substances in-field [11]. However, these instruments are only suitable for suspicious powder with high-purity unknown substances. The high accuracy, flexibility and efficiency of gas chromatography (GC) and liquid chromatography (LC) have made them the leading separation techniques in chromatography. GC and LC are capable of achieving the physical separation of multiple components in a mixture, and mass spectrometry (MS) provides information about the structure [12]. Additionally, due to its widespread use in many forensic laboratories worldwide, chromatography-mass spectrometry technology is the preferred analytical method for the analysis of unknown substances in the absence of reference standards. Liu et al. (2021) identified three new types of synthetic cannabinoids based on gas chromatography-mass spectrometry (GC-MS), liquid chromatography-mass spectrometry (LC-MS), NMR and FT-IR, which are beyond the class-wide ban of synthetic cannabinoids in China [13]. Fan et al. (2021) developed fragmentation patterns of synthetic cannabinoids based on electrospray ionization mass spectrometry and applied the fragmentation patterns to quickly screen the synthetic cannabinoids in electronic cigarette oil and tobacco from drug cases [14]. Qin et al. (2020) studied the fragmentation patterns of fentanyl analogues by high-resolution electron ionization mass spectrometry (EI-MS) and high-resolution electrospray ionization tandem mass spectrometry (ESI-MS), which could facilitate the detection and quantitation of fentanyl analogues [15]. The development speed of reference standards cannot keep up with the emergence speed of new analogues, and the prices of reference standards remain high due to the rapid evolution of the chemical structure of NPS [16]. Therefore, it is of great interest to develop MS fragmentation patterns to screen and structure identification of unknown substances, especially in mixtures.

Herein, eight ketamine analogues reference standards are systematically investigated by high-resolution gas chromatography-quadrupole time-of-flight mass spectrometry (GC-Q-TOF/MS) and liquid chromatography-quadrupole-orbitrap mass spectrometry (LC-Q-Orbitrap MS/MS). The fragmentation patterns of ketamine analogues are developed to facilitate forensic laboratories’ rapid screening of new substances with a similar structure. Additionally, evolutionary rules and fragmentation patterns are applied to detect and identify a new ketamine analogue in suspicious powder seized from a drug case. The structure of the new ketamine analogue is further confirmed by NMR. This is the first time that MS fragmentation patterns of ketamine analogues have been established and applied to the structure elucidation of new unknown ketamine analogue.

## 2. Results and Discussion

### 2.1. Overview of the Common Structure, EI-MS and ESI-MS/MS Fragmentation Patterns of Ketamine Analogues

Ketamine analogues belong to aromatic cyclohexanone amine derivatives, which are composed of an aryl ring, a cyclohexyl ring and an amine [17]. The chemical structure of common ketamine analogues, which is displayed in Scheme 1, is as follows: (i) The basic structure of ketamine analogues is 2-phenyl-2-aminocyclohexanone. (ii) The substituent R_1_ on aryl ring consists of a halogen atom (fluorine (F), chlorine (Cl) and bromine (Br)) or hydrogen atom (H). (iii) The substituent R_2_ on the aryl ring is made up of hydroxy (OH), methoxy (MeO) or a hydrogen atom (H). (iv) The substituent R_N1_ on amine group is mainly composed of an alkyl, such as methyl (CH_3_), ethyl (CH_2_CH_3_) and propyl (CH_2_CH_2_CH_3_).

### 2.2. Mass Spectra Results of Ketamine Analogues Reference Substances

Table 1 presents the compound structures, EI-MS and ESI-MS accurate and theoretical mass data for eight ketamine analogues reference substances, with deviation values essentially less than ±5 ppm. Table 2 exhibits the EI-MS and ESI-MS/MS data of eight ketamine analogue reference substances.

**Scheme 2:**
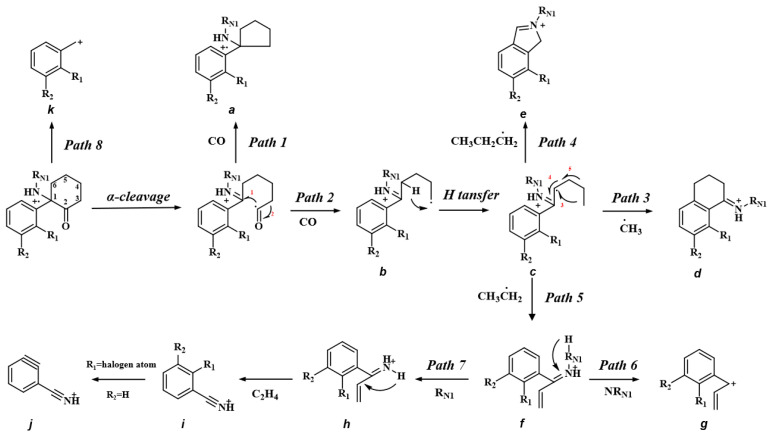
The common EI-MS fragmentation patterns of ketamine analogues.

The following are the common EI-MS fragmentation patterns (as shown in Scheme 2) on the basis of the GC-Q-TOF/MS analysis of eight ketamine analogue reference substances [18]: (i) The nitrogen atom of ketamine analogues is initially ionized via EI. (ii) Second, the α-cleavage of the carbon bond C_1_-C_2_ in the cyclohexanone moiety stabilizes the positive charge on the nitrogen atom, followed by the neutral loss of a CO (28 Da) and a five-membered ring formed to generate the stable fragment ***a*** via the fragmentation pathway 1 or the neutral loss of a CO (28 Da) to generate the unstable fragment ***b*** via the fragmentation pathway 2. (iii) The unstable fragment ***b*** is prone to causing hydrogen transfers to generate the fragment ***c***, leading to the radical approach to the positive nitrogen atom. (iv) Subsequently, fragment ***c*** loses a methyl radical (15 Da) and undergoes rearrangement to form a six-member ring to yield the fragment ***d*** via the fragmentation pathway 3. (v) Also, fragment ***c*** loses a propyl radical (43 Da) and undergoes rearrangement to form a five-member ring to generate the fragment ***e*** through the fragmentation pathway 4. (vi) The fragment ***f*** is generated by the loss of an ethyl radical (29 Da) from the fragment ***c*** based on the fragmentation pathway 5. (vii) The fragment ***f*** can further fragment into the ion ***g*** by losing an NR_N1_ according to the fragmentation pathway 6. NR_N1_ is often made of CH_3_N (29 Da), C_2_H_5_N (43 Da) and C_3_H_7_N (57 Da). (viii) Another fragmentation pathway of the fragment ***f*** yields the fragment ***h*** by loss of an R_N1_ (R_N1_ is usually composed of CH_2_ (14 Da), C_2_H_4_ (28 Da) and C_3_H_6_ (42 Da)) via the fragmentation pathway 7, further yielding the fragment ***i*** by the loss of a C_2_H_4_ (28 Da). (ix) When R_1_ and R_2_ are halogen and hydrogen atoms, respectively, fragment ***i*** can continue to lose halogenated hydrogen (HF (20 Da), HCl (36.5 Da), HBr (80 Da)) to yield the fragment ***j*** (*m*/*z* 104). (x). Ketamine analogues yield the fragment ***k*** by the cleavage of the carbon bond via the fragmentation pathway 8.

**Scheme 3:**
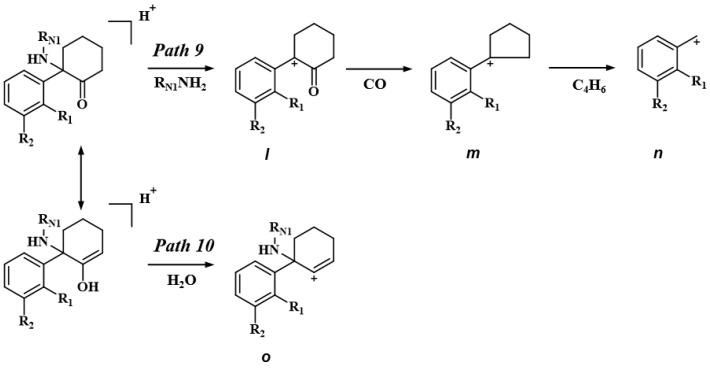
The common ESI-MS/MS fragmentation patterns of ketamine analogues in the positive ion mode.

The following are the common fragmentation patterns of ESI-MS/MS (illustrated in Scheme 3) according to a LC-Q-Orbitrap/MS analysis of eight ketamine analogue reference substances. (i) Ketamine analogues easily yield the characteristic fragments ***l*** and ***m*** by the loss of an R_N1_NH_2_ (R_N1_NH_2_ usually consists of CH_3_NH_2_ (31 Da), CH_3_CH_2_NH_2_ (49 Da) and CH_3_CH_2_CH_2_NH_2_ (42 Da)) and a CO (28 Da) through the fragmentation pathway 9 and further fragment into the fragment ***n*** by hydrogen transfer and the cleavage of the carbon bond. (ii) Another fragmentation pathway of ketamine analogues yields the fragment ***o*** by the loss of H_2_O (18 Da) via the fragmentation pathway 10.

### 2.3. Structure Elucidation of Ketamine Analogues Reference Substances

#### 2.3.1. 2-phenyl-2-(methylamino)cyclohexanone (DCK) and 2-(Ethylamino)-2-phenylcyclohexan-1-one (2-oxo-PCE)

**Scheme 4:**
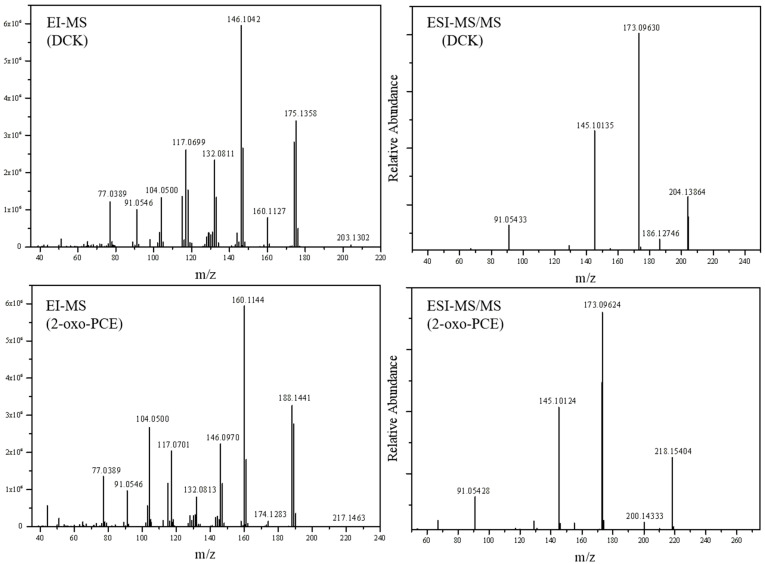
The EI-MS and ESI-MS/MS spectra of DCK and 2-oxo-PCE.

Scheme 4 shows the EI-MS and ESI-MS/MS spectra of DCK and 2-oxo-PCE. Data obtained from GC-Q/TOF high-resolution mass scanning showed that the molecule weights of DCK and 2-oxo-PCE are 203.1302 and 217.1463, respectively. The nitrogen atoms of DCK and 2-oxo-PCE are first ionized through EI, and then the α-cleavage takes place on the carbon bond C_1_-C_2_ in the cyclohexanone moiety of DCK and 2-oxo-PCE to stabilize the positive charge on the nitrogen atom. Subsequently, the ion at *m*/*z* 203.1302 of DCK yields the stable fragment at *m*/*z* 175.1357 by the neutral loss of a CO (28 Da) and a five-membered ring formed according to the fragmentation pathway 1 or generates the unstable fragment at *m*/*z* 175.1357 based on the fragmentation pathway 2 by neutral loss of a CO (28 Da). The ion at *m*/*z* 217.1463 of 2-oxo-PCE follows the same fragmentation pathways to produce the stable fragment ion and an unstable fragment ion, and the mass-to-charge ratio of these two fragments is 189.1518. The unstable fragment at *m*/*z* 175.1357 of DCK easily undergoes hydrogen transfer, allowing the radical to approach the ortho-nitrogen atom, further yielding the product ions at *m*/*z* 160.1127, *m*/*z* 146.1042 and *m*/*z* 132.08111 by the loss of methyl radical, ethyl radical and propyl radical based on the fragmentation pathways 3, 5 and 4, respectively. The unstable fragment at *m*/*z* 189.1518 of 2-oxo-PCE also produces fragments at *m*/*z* 174.1283, *m*/*z* 160.1144 and *m*/*z* 146.0970 in this sequence. The fragment at *m*/*z* 146.1042 of DCK continues to yield the fragments at *m*/*z* 132.0811 (loss of a CH_2_ via the fragmentation pathway 7), *m*/*z* 104.0500 (loss of a C_2_H_4_) and *m*/*z* 117.0699 (loss of a CH_3_N via the fragmentation pathway 6). The fragment at *m*/*z* 160.1144 of 2-oxo-PCE also obtains the same fragment ions at *m*/*z* 132.0813 (loss of a C_2_H_4_), *m*/*z* 104.0500 (loss of a C_2_H_4_) and *m*/*z* 117.0701 (loss of a C_2_H_5_N) via the same fragmentation pathways. These results suggest that substituents R_N1_ at the nitrogen atom were different. The substituents R_N1_ at the nitrogen atom of DCK and 2-oxo-PCE are methyl and ethyl, respectively. The fragments at *m*/*z* 91 and *m*/*z* 77, which are produced by DCK and 2-oxo-PCE, are the characteristic fragments of phenyl.

Data obtained from Q Orbitrap high-resolution mass scanning showed that the molecule weights of DCK and 2-oxo-PCE are 204.13843 and 218.15404, respectively. The HCD spectrums of DCK and 2-oxo-PCE show the fragments at *m*/*z* 173 (loss of a CH_3_NH_2_ from the DCK and loss of a CH_3_CH_2_NH_2_ from the 2-oxo-PCE based on the fragmentation pathway 9), *m*/*z* 145 (loss of a CO from the ion at *m*/*z* 173) and *m*/*z* 91 (loss of a C_4_H_6_ from the ion *m*/*z* 145). The protonated molecule of DCK at *m*/*z* 204.13843 and the protonated molecule of 2-oxo-PCE at *m*/*z* 218.15404 are prone to yielding the fragments at *m*/*z* 186.12746 and *m*/*z* 200.14333, respectively, by the neutral loss of H_2_O based on the classical fragmentation pathway 10. Schemes S1 and S2 show the EI-MS and ESI-MS/MS fragmentation pathways of DCK and 2-oxo-PCE.

#### 2.3.2. 2-(3-methoxyphenyl)-2-(ethylamino)cyclohexanone (MXE)

Data obtained from GC-Q/TOF high-resolution mass scanning shows that the molecule weight of MXE is 247.1567. The stabilized fragment ion at *m*/*z* 219.1619 is derived from the α-cleavage of the carbon bond C_1_-C_2_ on cyclohexanone, the loss of a CO and a five-membered ring formed from the molecular ion of MXE at *m*/*z* 247.1567 according to the fragmentation pathway 1. On the other hand, the unstabilized fragment ion at *m*/*z* 219.1619 arises from the α-cleavage of the carbon bond C_1_-C_2_ on cyclohexanone and the loss of a CO from the molecular ion of MXE at *m*/*z* 247.1567 according to the fragmentation pathway 2. Further losses of methyl radical, ethyl radical and propyl radical based on the fragmentation pathways 3, 5 and 4, respectively, yield the ions at *m*/*z* 204.1387, *m*/*z* 190.1301 and *m*/*z* 176.1095. The fragment at *m*/*z* 190.1301 yields the fragments at *m*/*z* 162.0915 (loss of a C_2_H_4_) and *m*/*z* 134.0603 (loss of a C_2_H_4_) following the fragmentation pathway 7. Also, the diagnostic fragment at *m*/*z* 147.0805 is produced via the fragmentation pathway 6 by losing a C_2_H_5_N from the ion at *m*/*z* 190.1301, confirming that the substituent R_N1_ on the amine group is ethyl. An additional featured fragmentation pathway of the molecular ion of MXE at *m*/*z* 247.1567 is mainly the cleavage of carbon bond to yield the fragment at *m*/*z* 121.0650, further fragmenting into the ions at *m*/*z* 91.0539 and *m*/*z* 77.0388 by sequential loss of HCHO and methyl radical. These results proved that the substituent R_1_ on the benzene ring of MXE is the methoxy group (-OCH_3_).

Data obtained from LC-Q/Orbitrap high-resolution mass scanning shows that the molecule weight of MXE is 248.16451. The HCD spectrum shows ions at *m*/*z* 203.10672 (loss of a CH_3_CH_2_NH_2_ from the protonated molecule of MXE at *m*/*z* 248.16451 via the fragmentation pathway 9), *m*/*z* 175.11182 (loss of a CO from ion at *m*/*z* 203.10672), *m*/*z* 121.06484 (loss of a C_4_H_6_ from ion at *m*/*z* 175.11182) and *m*/*z* 230.15408 (loss of a H_2_O from the protonated molecule of MXE at *m*/*z* 248.16451 via the fragmentation pathway 10). Schemes S3 and S4 show the EI-MS and ESI-MS/MS spectra and fragmentation pathways of MXE.

#### 2.3.3. 2-(2-Chlorophenyl)-2-(methylamino)cyclohexanone (Ketamine), 2-(2-Fluorophenyl)-2-(methylamino) cyclohexan-1-one (F-Ketamine), 2-(2-Bromophenyl)-2-(methylamino) cyclohexan-1-one (Br-Ketamine) and 2-(2-Chlorophenyl)-2-(ethylamino) cyclohexanone (NENK)

Data obtained from GC-Q/TOF high-resolution mass scanning shows that the molecule weights of ketamine, F-ketamine, Br-ketamine and NENK are 237.0913, 221.1205, 281.0423 and 251.1072, respectively. The α-cleavage of the carbon bond C_1_-C_2_ occurs in ketamine to produce the ion at *m*/*z* 237.0913. The additional loss of a CO from the ion at *m*/*z* 237.0913 forms the unstable ion at *m*/*z* 209.0965 or forms a five-membered ring from the ion at *m*/*z* 237.0913 to yield the stable product ion at *m*/*z* 209.0965. The unstable ion at *m*/*z* 209.0965 also easy yields product ions at *m*/*z* 194.0732 (loss of a methyl radical), *m*/*z* 180.0591 (loss of an ethyl radical) and *m*/*z* 132.0811 (loss of a propyl radical) based on the fragmentation pathways 3, 5 and 4, respectively. F-ketamine produces the ions at *m*/*z* 221.1205 (α-cleavage of the carbon bond C_1_-C_2_), *m*/*z* 193.1265 (loss of a CO or loss of a CO and formation of a five-membered ring), *m*/*z* 178.1034 (loss of a methyl radical), *m*/*z* 164.0942 (loss of an ethyl radical) and *m*/*z* 150.0718 (loss of a propyl radical) in this sequence. Br-ketamine and NENK also undergo the same fragmentation pathways to produce the ions at *m*/*z* 281.0423 (α-cleavage of the carbon bond C_1_-C_2_), *m*/*z* 253.0478 (loss of a CO or loss of a CO and formation of a five-membered ring), *m*/*z* 238.0219 (loss of a methyl radical), *m*/*z* 224.0092 (loss of an ethyl radical) and *m*/*z* 209.9915 (loss of a propyl radical) of Br-ketamine and the ions at *m*/*z* 251.1072 (α-cleavage of the carbon bond C_1_-C_2_), *m*/*z* 223.1125 (loss of a CO or loss of a CO and formation of a five-membered ring), *m*/*z* 208.0890 (loss of a methyl radical), *m*/*z* 180.0579 (loss of an ethyl radical) and *m*/*z* 194.0758 (loss of a propyl radical) of NENK. The ion at *m*/*z* 180.0591 of ketamine, the ion at *m*/*z* 164.0942 of F-ketamine and the ion at *m*/*z* 224.0092 of Br-ketamine yield the fragments at *m*/*z* 166.0418, *m*/*z* 150.0718 and *m*/*z* 209.9915 by the loss of a CH_2_, further losing a C_2_H_4_ to produce the fragments at *m*/*z* 138.0105, *m*/*z* 122.0406 and *m*/*z* 181.9616, respectively. Also, the ions at *m*/*z* 151.0312, *m*/*z* 135.0606 and *m*/*z* 194.9801 are produced by the loss of a CH_3_N from the ion at *m*/*z* 180.0591 of ketamine, the ion at *m*/*z* 164.0942 of F-ketamine and the ion at *m*/*z* 224.0092 of Br-ketamine, respectively. However, the ion at *m*/*z* 194.0758 of NENK yields the fragments at *m*/*z* 166.0423 (loss of a C_2_H_4_) and *m*/*z* 151.0312 (loss of a C_2_H_4_N). And the ion at *m*/*z* 166.0423 can further generate the ion at *m*/*z* 138.0108 through the loss of a C_2_H_4_. Based on the above information, the substituents R_N1_ on the amino group of ketamine, F-Ketamine and Br-Ketamine are methyl, and that of NENK is ethyl. Moreover, the ions at *m*/*z* 125.0151, *m*/*z* 109.0542, *m*/*z* 168.9652 and *m*/*z* 125.0156, which are produced by Ketamine, F-Ketamine, Br-Ketamine and NENK, respectively, via the fragmentation pathway 8. The further loss of HF, HCl or HBr yields the same ion at *m*/*z* 75. Additionally, the common ions at *m*/*z* 105 and *m*/*z* 102 can similarly elucidate the halogen substituents on the benzene ring of ketamine analogues.

Data obtained from LC-Q/Orbitrap high-resolution mass scanning shows that the molecule weights of ketamine, F-ketamine, Br-ketamine and NENK are 238.09952, 222.12936, 282.04880 and 252.11505, respectively. A neutral loss of H_2_O from the protonated molecule of ketamine, F-ketamine, Br-ketamine and NENK fragments into the ions at *m*/*z* 220.08894, *m*/*z* 204.11887, *m*/*z* 264.03821 and *m*/*z* 234.10452, respectively, via the fragmentation pathway 10. Additionally, the ions at *m*/*z* 207.05742, *m*/*z* 179.06247 and *m*/*z* 125.01547 of ketamine, the ions at *m*/*z* 191.08719, *m*/*z* 163.09216 and *m*/*z* 109.04510 of F-ketamine and the ions at *m*/*z* 251.00664, *m*/*z* 223.01180 and *m*/*z* 168.96486 of Br-ketamine are prone to yielding by losing a CH_5_N, a CO and a C_4_H_6_, respectively, via the fragmentation pathway 9. On the other hand, NENK is apt to produce the ions at *m*/*z* 207.05725, *m*/*z* 179.06230, and *m*/*z* 125.01538 by losing a C_2_H_7_N, a CO and a C_4_H_6_, respectively, via the same fragmentation pathway 9. The common ions at *m*/*z* 207 of ketamine and NENK can further yield the ions at *m*/*z* 163 by the loss of a C_2_H_4_O. Another fragmentation pathway of Br-ketamine concerns the loss of an HBr and a CH_2_O to generate the ion at *m*/*z* 172.08832. The findings of the LC-Q/Orbitrap high-resolution mass scanning can further validate that the substituents R_N1_ on the amino group of ketamine, F-ketamine and Br-ketamine are methyl and the substituent R_N1_ of NENK is ethyl. Schemes S5–S12 show the EI-MS and ESI-MS/MS fragmentation pathways of ketamine, F-ketamine, Br-ketamine and NENK.

#### 2.3.4. 2-(Ethylamino)-2-(m-tolyl)cyclohexan-1-one (DMXE)

Data obtained from GC-Q/TOF high-resolution mass scanning shows that the molecule weight of DMXE is 231.1617. The molecular ion at *m*/*z* 231.1617 fragments into the stable ion at *m*/*z* 203.1668 and the unstable ion at *m*/*z* 203.1668 via the fragmentation pathways 1 and 2, respectively. The fragmentation pathway of the unstable ion at *m*/*z* 203.1668 concerns the loss of methyl radical, ethyl radical and propyl radical, leading to the ions at *m*/*z* 188.1438, *m*/*z* 174.1284 and *m*/*z* 160.1124 based on the fragmentation pathways 3, 5 and 4, respectively. The diagnostic ion at *m*/*z* 131.0855 can be attributed to the loss of C_2_H_5_N from the ion at *m*/*z* 174.1284, revealing that the substituent R_N1_ of DMXE is ethyl. The ion at *m*/*z* 174.1284 further yields the ions at *m*/*z* 146.0967 and *m*/*z* 118.0654 by consecutive loss of two C_2_H_4_. The ions at *m*/*z* 105, *m*/*z* 91 and *m*/*z* 77 are produced by DMXE via the fragmentation pathway 8.

Data obtained from LC-Q/Orbitrap high-resolution mass scanning shows that the molecule weight of DMXE is 232.16963. The protonated molecule at *m*/*z* 232.16963 easily yields fragments at *m*/*z* 187.11192 (loss of a CH_5_N from the ion at *m*/*z* 232.16963), *m*/*z* 159.11699 (loss of a CO from the ion at *m*/*z* 187.11192), *m*/*z* 105.07034 (loss of a C_4_H_6_ from the ion at *m*/*z* 159.11699)and *m*/*z* 214.15903 (loss of a H_2_O from the protonated molecule at *m*/*z* 232.16963) following the typical fragmentation pathways 9 and 10. Schemes S13 and S14 show the EI-MS and ESI-MS/MS spectra and fragmentation pathways of DMXE.

### 2.4. Analysis of Mass Spectrometry Fragmentation Patterns of Ketamine Analogues

With the comparison of the EI-MS and ESI-MS/MS fragmentation patterns of ketamine analogues, the characteristic ions ***a*** (α-cleavage, loss of a CO and form a five-member ring, 28 Da), ***c*** (α-cleavage and loss of a CO, 28 Da), ***d*** (loss of a methyl radical, 15 Da), ***f*** (loss of an ethyl radical, 29 Da) and ***e*** (loss of a propyl radical, 43 Da) can be susceptible to generation via the fragmentation pathways 1, 2, 3, 5 and 4, respectively, in EI-MS mode, which can be used to rapidly identify the basic structure of ketamine analogues. On the other hand, in ESI-MS/MS mode, the diagnostic ions ***m*** (loss of a CO), ***n*** (loss of a C_4_H_6_) and ***o*** (loss of a H_2_O) can be easily produced via the fragmentation pathways 9 and 10, which can also be employed to rapidly infer the basic structure of the ketamine analogs quickly. The ions ***h*** and ***g*** generated via the fragmentation pathways 6 and 7 in EI-MS mode and the ion ***l*** produced via the fragmentation pathway 9 in ESI-MS/MS mode are employed to confirm the structure of substituent R_N1_ on the amino group. Also, when the substituent R_1_ is -F, -Cl or -Br, the ion ***i*** can undergo further cleavage to lose an HF, HCl or HBr, thus determining the structure of substituent R_1_.

## 3. Qualitative Analysis of Suspicious Powder

**Scheme 5:**
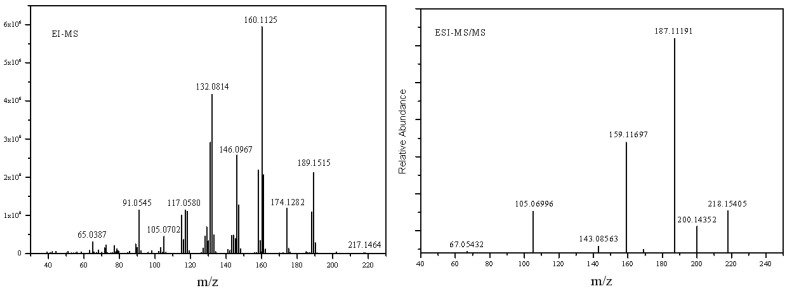
The EI-MS and ESI-MS/MS spectra of compound **1**.

**Scheme 6:**
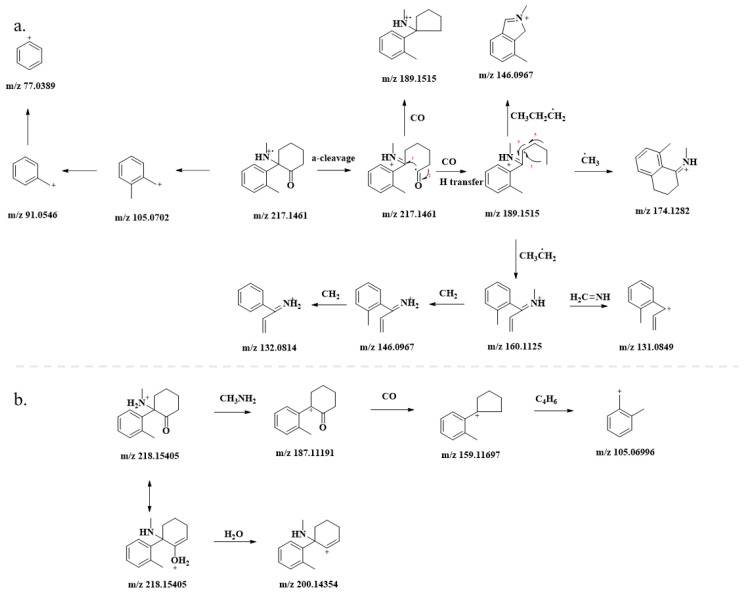
(**a**). The EI-MS, and (**b**). ESI-MS/MS fragmentation pathways of compound **1**.

Scheme 5 exhibits the EI-MS and ESI-MS/MS spectra of compound **1**. Table 3 shows the accurate mass numbers of the molecular ion, the protonated molecular and predominant product ions and their proposed chemical formula obtained for the suspicious powder measured by EI-QTOF/MS and ESI-Q-Orbitrap MS/MS, and the deviation values are approximately under ±5 ppm. The EI-QTOF/MS displays the molecular ion at *m*/*z* 217.1461 (C_14_H_19_NO^+^), and the ESI-Q-Orbitrap/MS shows the protonated ion at *m*/*z* 218.15405 (C_14_H_20_NO^+^). The mass difference between compound **1** and a known compound DCK is 14 Da (CH_2_), which indicates that compound **1** in suspicious powder has an additional methyl moiety compared with DCK. The molecular ion at *m*/*z* 217.1461 yields fragments at *m*/*z* 189.1515 (α-cleavage, loss of a CO and formation of a five-member ring), *m*/*z* 189.1515 (α-cleavage and loss of a CO), *m*/*z* 174.1282 (loss of a methyl radical), *m*/*z* 160.1125 (loss of an ethyl radical) and *m*/*z* 146.0967 (loss of a propyl radical) based on the fragmentation pathways 1, 2, 3, 5 and 4, respectively, in EI-MS mode. Moreover, the HCD spectrum exhibits the characteristic ions at *m*/*z* 200.14354 (loss of a H_2_O), 159.11697 (loss of a CO) and *m*/*z* 105.06996 (loss of a C_4_H_6_) through the fragmentation pathways 9 and 10 in ESI-MS/MS mode. These results indicate that the basic structure of compound **1** is 2-phenyl-2-aminocyclohexanone, and compound **1** belongs to the ketamine analogues. The ions at *m*/*z* 131.0849 (loss of a CH_3_N), *m*/*z* 146.0967 (loss of a CH_2_) and *m*/*z* 132.0814 (loss of a CH_2_) are produced by the ion at *m*/*z* 160.1125 based on the fragmentation pathways 6 and 7, respectively, in EI-MS mode, and the ion at *m*/*z* 187.11191 (loss of a CH_3_NH_2_) is generated by the protonated molecule at *m*/*z* 218.15405 via the fragmentation pathway 9 in ESI-MS/MS mode, indicating that the structure of substituent R_N1_ is methyl. Also, the molecular ion at *m*/*z* 217.1461 yields fragments at *m*/*z* 105.0702 (the cleavage of carbon bond), *m*/*z* 91.0546 (loss of a CH_2_) and *m*/*z* 77.0389 (loss of a CH_2_) via the fragmentation pathway 8 in EI-MS mode. In ESI-MS/MS mode, the difference in the chemical formula between the ion at *m*/*z* 105.06996 (C_8_H_9_^+^) produced by compound **1** and the ion at 91.05479 (C_7_H_7_^+^) generated by DCK is an extra CH_2_. These results confirm that compound **1** has an additional methyl on the benzene ring. Scheme 5 and Scheme 6 show the EI-MS and ESI-MS/MS spectra and fragmentation pathways of compound **1**. As a consequence of the above information, the structure of compound **1** is reasonably inferred to be 2-(Methylamino)-2-(o-tolyl)cyclohexan-1-one or 2-(Methylamino)-2-(m-tolyl)cyclohexan-1-one.

The structure of compound **1** is further elucidated by NMR. The ^1^H NMR spectra (Scheme S15) of this compound suggest that there are two substituents on the benzene ring [ArH (δ_H_ 7.74, 1H, m), ArH (δ_H_ 7.45, 2H, m), ArH (δ_H_ 7.39, 1H, m)]. Moreover, the ArH (δ_H_ 7.74), ArH (δ_H_ 7.45) and ArH (δ_H_ 7.39) in the ^1^H/^1^H-COSY (Scheme S16) of this compound are correlated, indicating that the two substituents on the benzene ring are located in adjacent positions. Therefore, compound **1** is 2-(Methylamino)-2-(o-tolyl)cyclohexan-1-one.

## 4. Materials and Methods

### 4.1. Materials

Methanol and acetonitrile (HPLC grade) were obtained from Merck Chemical (Darmstadt, Germany). The reference standards of 2-phenyl-2-(methylamino)cyclohexanone (DCK), 2-(Ethylamino)-2-phenylcyclohexan-1-one (2-oxo-PCE), 2-(3-methoxyphenyl)-2-(ethylamino)cyclohexanone (MXE), 2-(2-Chlorophenyl)-2-(methylamino) cyclohexanone (Ketamine), 2-(2-Fluorophenyl)-2-(methylamino) cyclohexan-1-one (F-Ketamine), 2-(2-Bromophenyl)-2-(methylamino)cyclohexan-1-one (Br-Ketamine), 2-(2-Chlorophenyl)-2-(ethylamino) cyclohexanone (NENK), 2-(Ethylamino)-2-(m-tolyl)cyclohexan-1-one (DMXE) were obtained from Shanghai Yuansi Standard Science and Technology Co., Ltd. Suspicious powder was seized from drug cases. And water was purified by a Millipore Milli-Q-Gradient purification system.

### 4.2. Instrument

#### 4.2.1. GC-Q-TOF/MS Analysis

GC-Q-TOF/MS is a combination of an Agilent 8890 GC and an Agilent 7250 Q-TOF/MS (Agilent, Santa Clara, CA, USA). The conditions of GC were as follows: capillary column is HP-5ms (30 m length, 250 μm i.d, 0.25 μm film thickness, Agilent, Santa Clara, CA, USA). The carrier gas was high helium (purity 99.999%) with a constant flow rate of 1.0 mL·min^−1^. The inlet temperature was 275 °C. The oven temperature program was initiated at 60 °C and then raised to 280 °C at a rate of 20 °C·min^−1^ for 11 min. The injection volume was 1 μL with an injection split ratio of 10:1. The conditions of MS are as follows: ionization mode was EI (70 eV), and the acquisition range was 50 to 550 *m*/*z* in full-scan mode. The temperature of the ion source and quadrupole were 150 °C and 230 °C, respectively. GC-Q-TOF/MS control, peak integration (peak areas were integrated into total ion chromatogram) and mass spectra evaluation were performed using Qualitative Analysis Software 10.0 (Agilent, Santa Clara, CA, USA).

#### 4.2.2. LC-Q-Orbitrap/MS Analysis

A Thermo Scientific Dionex Ultimate 3000 system (Thermo Fisher Scientific, Waltham, MA, USA) with a Hypersil GOLD VANQUISH (100 × 2.1 mm, 1.9 μm) column was coupled with a Thermo Scientific Q Exactive mass spectrometer (Thermo Fisher Scientific, Waltham, MA, USA) with positive heated electrospray spraying ionization mode (HESI^+^). The mobile phase, which was made up of acetonitrile (solvent A) and 1% formic acid in water (solvent B), was run in gradients: 0–2 min, 95% B; 2–9.5 min, 95–5% B 9.5–12 min 5% B; 12.1 min, 95% B. The flow rate was 0.3 mL·min^−1^, and the injection volume was 2 μL.

The parameters of MS wereeas follows: collision gas: nitrogen; capillary temperature: 350 °C; spray voltage: 3800 V; auxiliary gas pressure: 15 arb; nebulizer pressure: 35 arb; transmission capillary temperature: 350 °C. Mass resolution used was 14,000; maximum infusion time (IT): 50 ms; automatic gain control (AGC) target: 3 × 10^6^. Full MS-ddMS^2^ was used with scan range: 160–600 amu; higher energy collision dissociation (HCD) was 25 NCE; AGC target: 1 × 10^5^; mass resolution: 35,000; isolation window: 4 amu; maximum IT: 50 ms. Control of the instrument and data processing were carried out using XCalibur 4.0 software (Thermo Scientific, Waltham, MA, USA). We simulated and studied the fragmentation behavior of the described compounds using Mass Frontier 7.0 software (Thermo Finnigan, Waltham, MA, USA).

### 4.3. Sample Preparation

Preparation of eight ketamine analogue reference substances solution for GC-Q-TOF/MS and LC-Q-Orbitrap/MS: each of ketamine analogue reference substances were dissolved in methanol at a concentration of 100 ng·L^−1^.

Preparation of sample solutions: we transferred 1 mg of powder into 10 mL centrifuge tube and then added 10 mL methanol, ultrasoniced until mixed well and filtered through a syringe filter (0.22 μm).

## 5. Conclusions

Eight ketamine analogues were systematically investigated by GC-Q-TOF/MS and LC-Q-Orbitrap/MS with the positive mode of electrospray ionization. Since these substances have the same skeleton and similar molecular structures, the fragmentation patterns and fragments also have a high degree of similarity in EI-MS and ESI-MS/MS modes. The mass spectrometry fragmentation patterns of ketamine analogues are deduced based on the high-resolution MS data. The fragment ions a (α-cleavage, loss of CO and formation of a five-member ring, 28 Da), ***c*** (α-cleavage, H transfer and loss of a CO, 28 Da), ***d*** (loss of a methyl radical, 15 Da), ***f*** (loss of an ethyl radical, 29 Da) and ***e*** (loss of a propyl radical, 43 Da) in EI-MS mode and the fragment ions ***m*** (loss of a CO, 28 Da), ***n*** (loss of a C_4_H_6_, 54 Da) and ***o*** (loss of an H_2_O, 18 Da) in ESI-MS/MS mode are characteristic ions of ketamine analogues. The fragment ions ***a****, **c**, **d**, **e**, **f**, **k**, **o**, **m*** and ***n*** can be used for the rapid identification of the basic structures of ketamine analogues. And fragment ions ***h****, **g**, **k*** and ***l*** can be applied for confirmation of the substituents of ketamine analogue. In addition, the structure of suspicious substances from drug cases is deduced based on the MS fragmentation patterns and evolutionary rules of ketamine analogues and novel ketamine analogues. This study could enable the rapid structure identification of ketamine analogues, which would be useful to assist forensic laboratories in identifying such compounds.

## Figures and Tables

**Table 1:** Compound structures, EI-MS and ESI-MS accurate and theoretical mass data and RSD of eight ketamine analogue reference substances.

No.	Compound Structures	Formula	Accurate *m*/*z* ([M^+^])	Theoretical *m*/*z* ([M^+^])	Accurate *m*/*z* ([M + H]^+^)	Theoretical *m*/*z* ([M + H]^+^)	RSD (ppm) *^a^*
1	DCK: 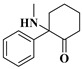	C_13_H_17_NO	203.1302	203.1310	204.13843	204.13829	−3.94/0.69
2	2-oxo-PCE: 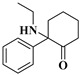	C_14_H_19_NO	217.1463	217.14666	218.15404	218.15394	−1.65/0.46
3	MXE: 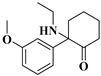	C_15_H_21_NO_2_	247.1567	247.15723	248.16451	248.16451	−2.14/0
4	Ketamine: 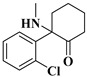	C_13_H_16_ClNO	237.0913	237.09204	238.09952	238.09932	−3.12/0.84
5	F-Ketamine: 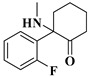	C_13_H_16_FNO	221.1205	221.12159	222.12936	222.12887	−4.93/2.20
6	Br-Ketamine: 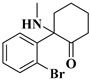	C_13_H_16_BrNO	281.0423	281.04153	282.04880	282.04880	2.73/0
7	NENK: 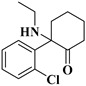	C_14_H_18_ClNO	251.1072	251.10769	252.11505	252.11497	−1.95/0.32
8	DMXE: 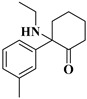	C_15_H_21_NO	231.1617	231.16231	232.16963	232.16959	−2.64/0.17

*^a^* Relative standard deviation (RSD) is calculated as [(*M* − *M*_0_)/*M*_0_] × 10^6^ (ppm).

**Table 2:** EI-MS and ESI-MS/MS fragmentation ions of eight ketamine analogues reference substances.

**Comp.**	**Molecular Ion (*m*/*z*)**	**Fragmentation Ions (*m*/*z*)**
**EI-MS**		
**1**	203.1302	175.1358 (Path 1, 2), 160.1127 (Path 3), 146.1042 (Path 5), 132.0811 (Path 4, 7), 117.0699 (Path 6), 104.0500 (Path 7), 91.0546 (Path 8), 77.0389 (Path 8)
**2**	217.1463	189.1518 (Path 1, 2), 174.1283 (Path 3), 160.1144 (Path 5), 146.0970 (Path 4), 132.0813 (Path 7), 117.0701 (Path 6), 104.0500 (Path 7), 91.0546 (Path 8), 77.0389 (Path 8)
**3**	247.1567	219.1619 (Path 1, 2), 204.1387 (Path 3), 190.1301 (Path 5), 176.1085 (Path 4), 162.0915 (Path 7), 147.0805 (Path 6), 134.0603 (Path 7), 121.0650 (Path 8),91.0539 (Path 8), 77.0388 (Path 8)
**4**	237.0913	209.0965 (Path 1, 2), 194.0732 (Path 3), 180.0591 (Path 5), 166.0418 (Path 7), 145.0885 (Path 1) 138.0105 (Path 7), 125.0151 (Path 8), 115.0542 (Path 6), 102.0341 (Path 7), 75.0229 (Path 8)
**5**	221.1205	193.1265 (Path 1, 2), 178.1034 (Path 3), 164.0942 (Path 5), 150.0718 (Path 4, 7), 135.0606 (Path 6), 122.0406 (Path 7), 115.0547 (Path 6), 109.0542 (Path 8), 102.0345 (Path 7), 75.0232 (Path 8)
**6**	281.0423	253.0480 (Path 1, 2), 238.0219 (Path 3), 224.0092 (Path 5), 209.9915 (Path 4, 7), 194.9801 (Path 6), 181.9616 (Path 7), 168.9652 (Path 8), 145.0900 (Path 1), 115.0553 (Path 6), 102.0470 (Path 7), 75.0229 (Path 8)
**7**	251.1072	223.1125 (Path 1, 2), 208.0890 (Path 3), 194.0758 (Path 5), 180.0579 (Path 4), 166.0423 (Path 7), 151.0312 (Path 6), 138.0108 (Path 7), 125.0156 (Path 8), 115.0545, 102.0342 (Path 8), 75.0232 (Path 8)
**8**	231.1617	203.1669 (Path 1, 2), 188.1438 (Path 3), 174.1284 (Path 5), 160.1124 (Path 4), 146.0967 (Path 7), 131.0855 (Path 6), 118.0654 (Path 7), 105.0701 (Path 8), 91.0544 (Path 8), 77.0387 (Path 8)
**Comp.**	**Precursor Ion (*m*/*z*)**	**Fragmentation Ions (*m*/*z*)**
**ESI-MS/MS**		
**1**	204.13843	186.12746 (Path 10), 173.09630 (Path 9), 145.10135 (Path 9), 91.05433 (Path 9)
**2**	218.15404	200.14333 (Path 10), 173.09624 (Path 9), 145.10124 (Path 9), 91.05428 (Path 9)
**3**	248.16451	230.15408 (Path 10), 203.10672 (Path 9), 175.11182 (Path 9), 121.06484 (Path 9)
**4**	238.09952	220.08894 (Path 10), 207.05742 (Path 9), 179.06247 (Path 9), 125.01547 (Path 9), 163.03113
**5**	222.12936	204.11887 (Path 10), 191.08719 (Path 9), 163.09216 (Path 9), 109.04510 (Path 9)
**6**	282.04880	264.03821 (Path 10), 251.00664 (Path 9), 223.01180 (Path 9), 168.96486 (Path 9), 172.08832
**7**	252.11505	234.10452 (Path 10), 207.05725 (Path 9), 179.06230 (Path 9), 125.01538 (Path 9), 163.03096
**8**	232.16963	214.15903 (Path 10), 187.11192 (Path 9), 159.11699 (Path 9), 105.07034 (Path 9)

**Table 3:** Accurate mass numbers of the molecular ion, the protonated ion and predominant product ions and their proposed chemical formulas obtained for the suspicious powder measured by EI-QTOF/MS and ESI-Q-Orbitrap MS/MS.

EI-QTOF/MS				
Compound	Chemical Formula	Exact Mass	Accurate Mass	Error (ppm)
**1**	C_14_H_19_NO^+^	217.1461	217.1461	0.0
	C_13_H_19_N^+^	189.1512	189.1515	1.6
	C_12_H_16_N^+^	174.1277	174.1282	2.9
	C_11_H_14_N^+^	160.1121	160.1125	2.5
	C_10_H_12_N^+^	146.0964	146.0967	2.1
	C_9_H_10_N^+^	132.0808	132.0814	4.5
	C_10_H_11_^+^	131.0855	131.0849	−4.6
	C_8_H_9_^+^	105.0699	105.0702	2.9
	C_7_H_7_^+^	91.0542	91.0546	4.4
	C_6_H_5_^+^	77.0386	77.0389	3.9
ESI-Q-Orbitrap MS/MS				
**1**	C_14_H_20_NO^+^	218.15394	218.15405	0.5
	C_14_H_18_N^+^	200.14338	200.14354	0.8
	C_13_H_15_O^+^	187.11174	187.11191	0.9
	C_12_H_15_^+^	159.11683	159.11697	0.9
	C_8_H_9_^+^	105.06988	105.06996	4.7

## Data Availability

All the data are available within the manuscript.

## Supplementary Materials

The following supporting information can be downloaded at: https://www.mdpi.com/article/10.3390/molecules28186510/s1, Scheme S1: (a) The EI-MS and (b) ESI-MS/MS fragmentation pathways of DCK; Scheme S2: (a) The EI-MS and (b) ESI-MS/MS fragmentation pathways of 2-oxo-PCE; Scheme S3: The EI-MS and ESI-MS/MS spectra of MXE; Scheme S4: (a) The EI-MS and (b) ESI-MS/MS fragmentation pathways of MXE; Scheme S5: The EI-MS and ESI-MS/MS spectra of ketamine; Scheme S6: (a) The EI-MS and (b) ESI-MS/MS fragmentation pathways of ketamine; Scheme S7: The EI-MS and ESI-MS/MS spectra of F-ketamine; Scheme S8: (a) The EI-MS and (b) ESI-MS/MS fragmentation pathways of F-ketamine; Scheme S9: The EI-MS and ESI-MS/MS spectra of Br-ketamine; Scheme S10: (a) The EI-MS and (b) ESI-MS/MS fragmentation pathways of Br-ketamine; Scheme S11: The EI-MS and ESI-MS/MS spectra of NENK; Scheme S12: (a) The EI-MS and (b) ESI-MS/MS fragmentation pathways of NENK; Scheme S13: The EI-MS and ESI-MS/MS spectra of DMXE; Scheme S14: (a) The EI-MS and (b) ESI-MS/MS fragmentation pathways of DMXE; Scheme S15: The 1H NMR spectra of compound **1**; Scheme S16: The 1H/1H COSY spectra of compound **1**.
