# Supplementary material for: Study on the Mass Spectrometry Fragmentation Patterns for Rapid Screening and Structure Identification of Ketamine Analogues in Illicit Powders
Source: Molecules. 2023 Sep 8;28(18):6510. doi: 10.3390/molecules28186510 (PMC10535375; doi:10.3390/molecules28186510)
Supplement: Supplementary file 1 [file molecules-28-06510-s001.zip › molecules-2569327-supplementary.pdf]

Supporting information

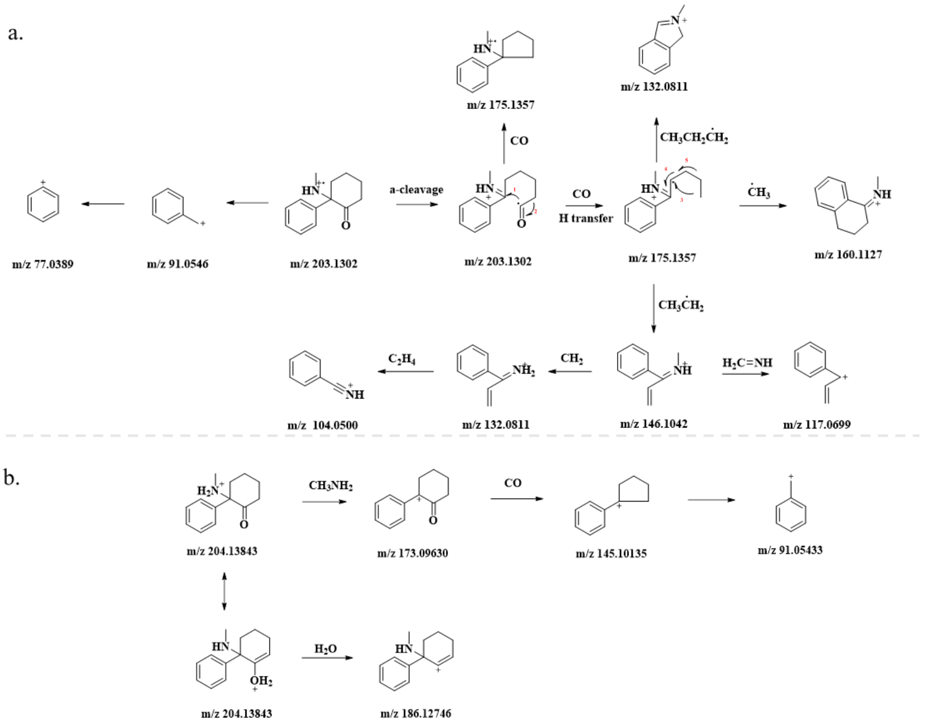

Scheme S1. a). The EI-MS and b). ESI-MS/MS fragmentation pathways of DCK.

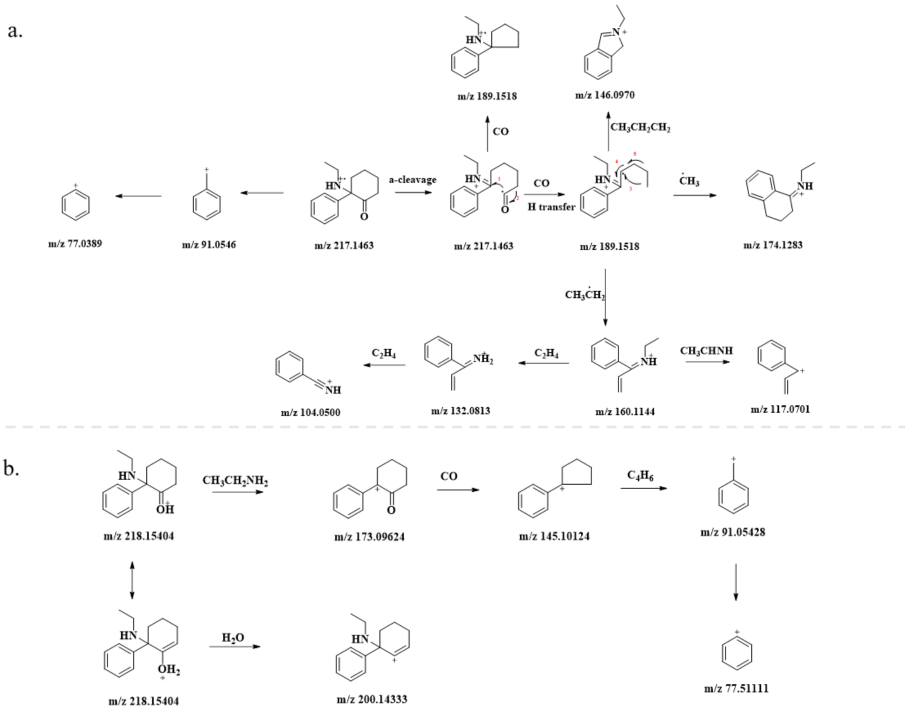

Scheme S2. a). The EI-MS and b). ESI-MS/MS fragmentation pathways of 2-oxo-PCE.

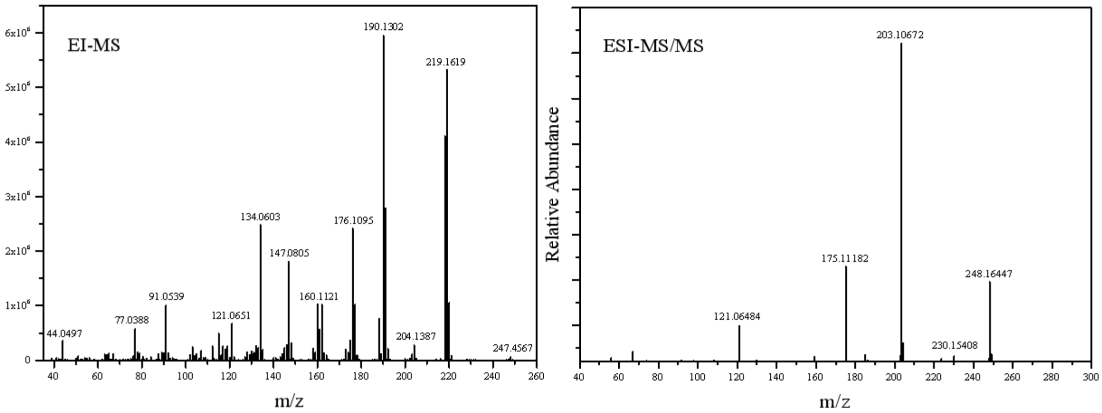

Scheme S3. The EI-MS and ESI-MS/MS spectra of MXE.

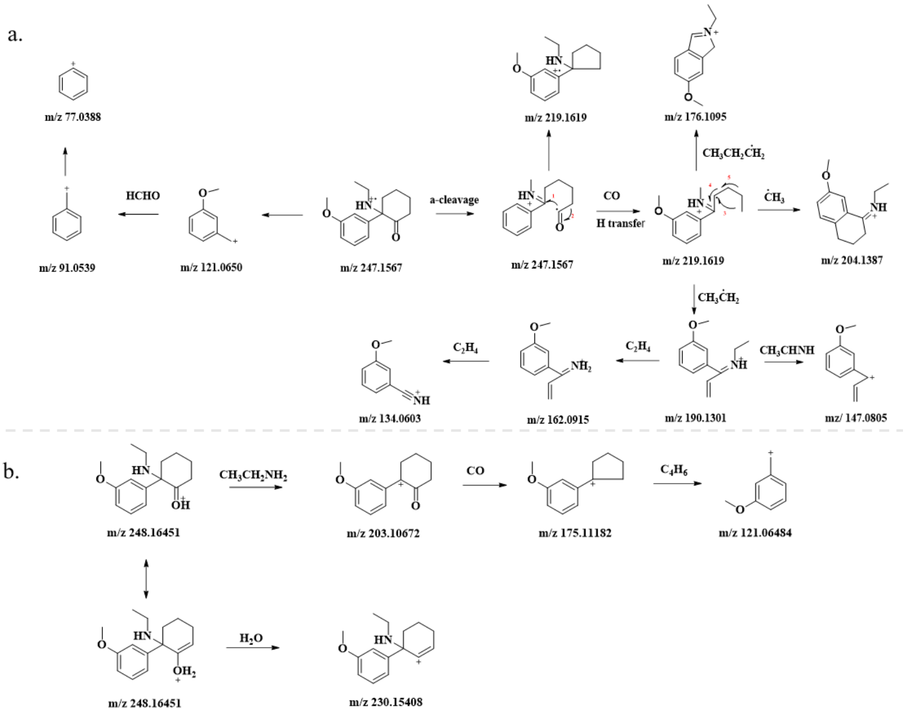

Scheme S4. a). The EI-MS and b). ESI-MS/MS fragmentation pathways of MXE.

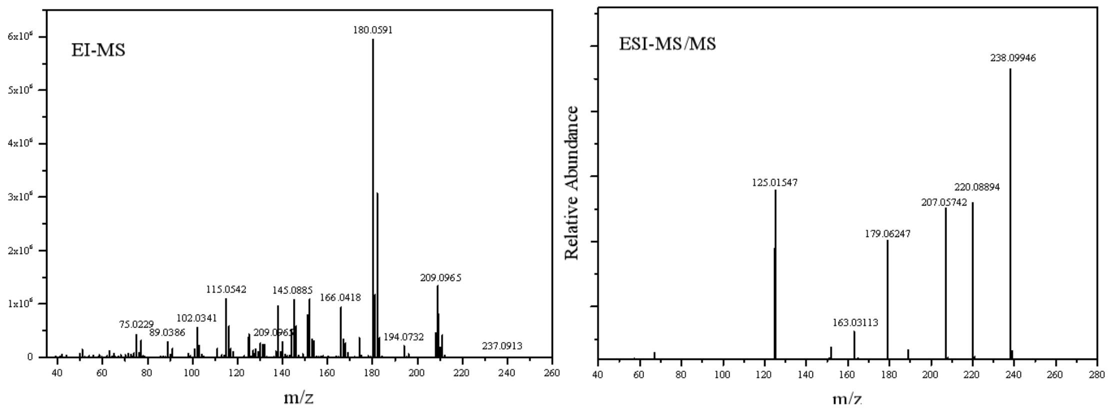

Scheme S5. The EI-MS and ESI-MS/MS spectra of ketamine.

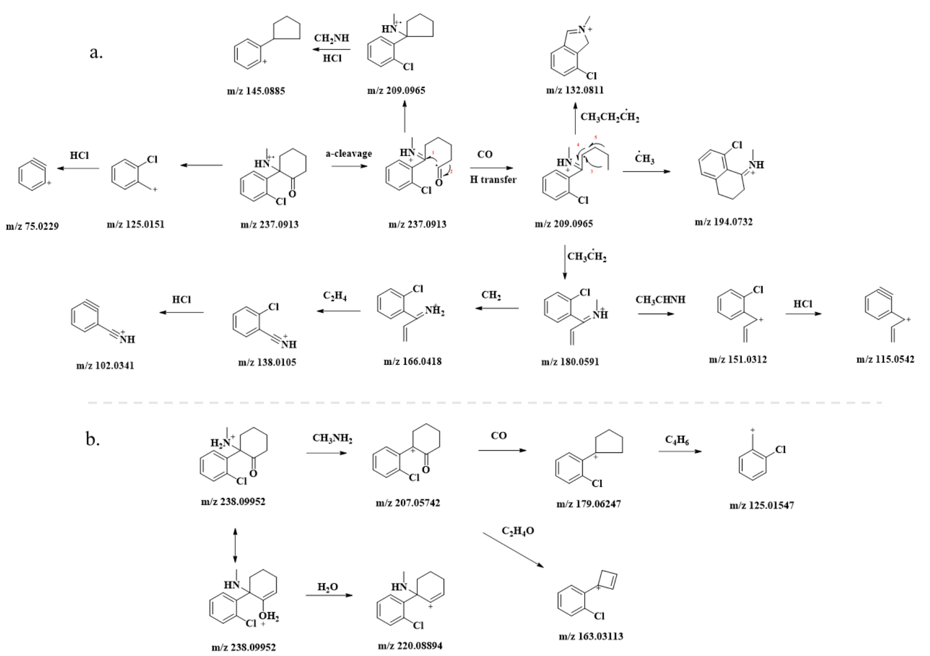

Scheme S6. a). The EI-MS and b). ESI-MS/MS fragmentation pathways of ketamine.

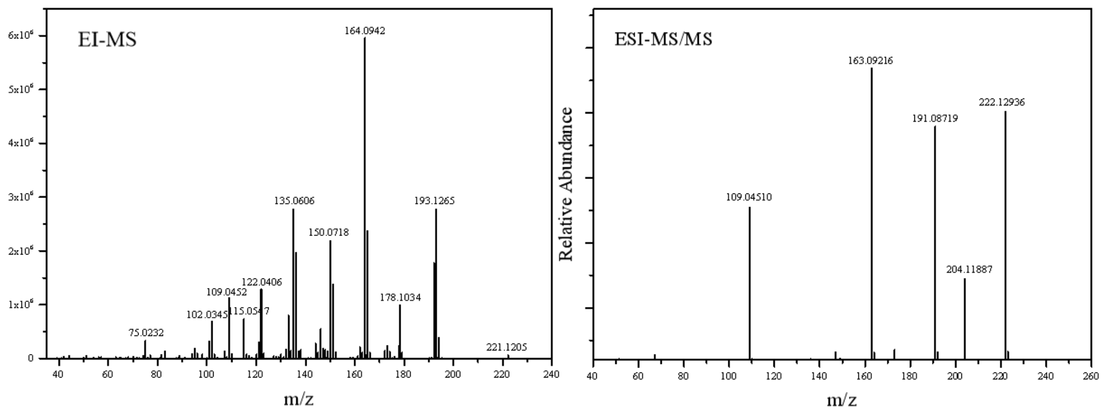

Scheme S7. The EI-MS and ESI-MS/MS spectra of F-ketamine.

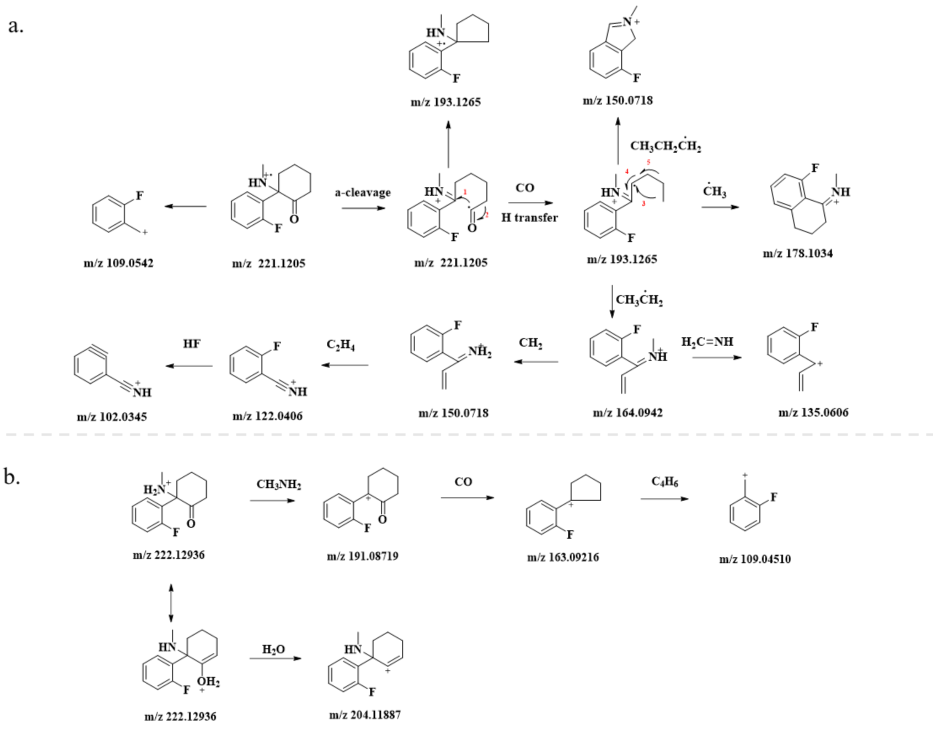

Scheme S8. a). The EI-MS and b). ESI-MS/MS fragmentation pathways of F-ketamine.

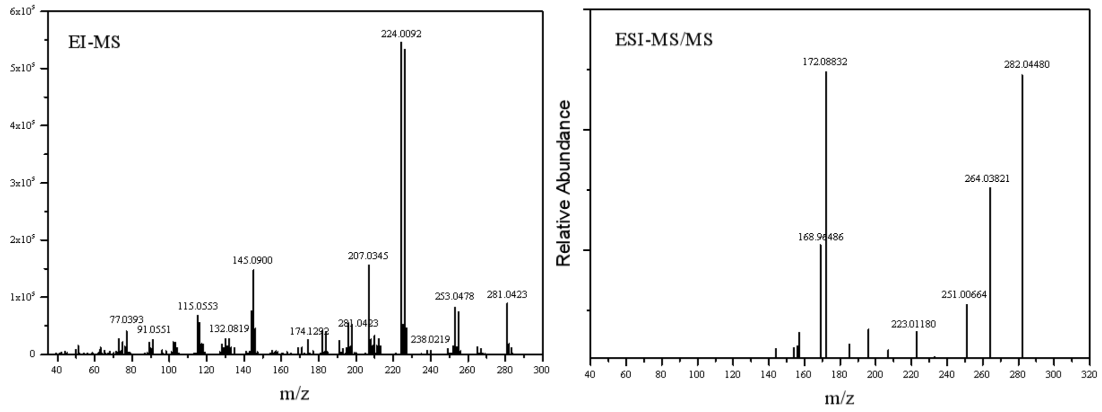

Scheme S9. The EI-MS and ESI-MS/MS spectra of Br-ketamine.

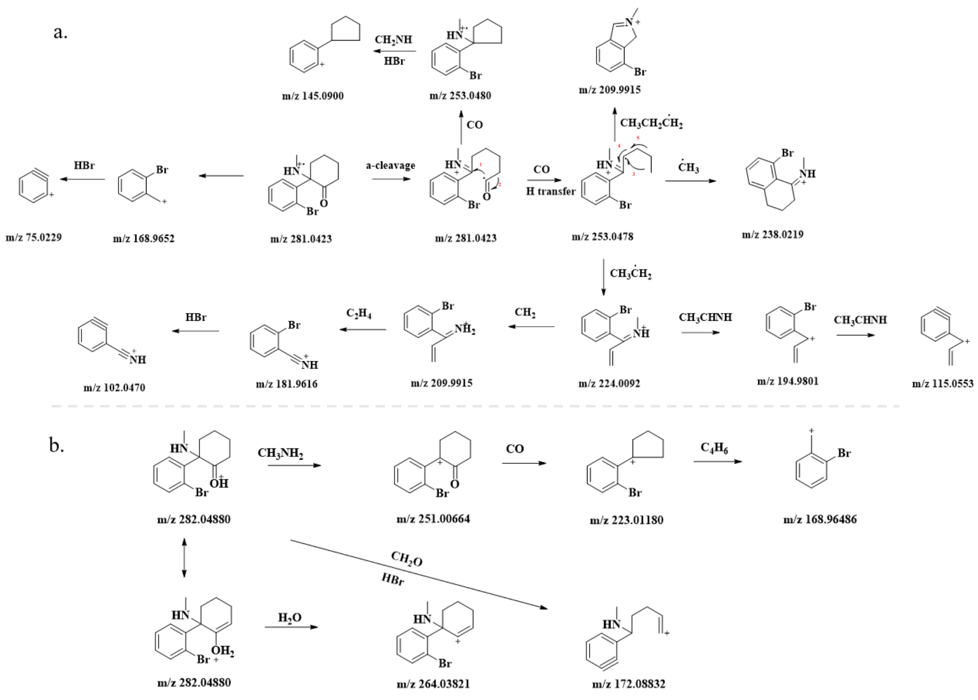

Scheme S10. a). The EI-MS and b). ESI-MS/MS fragmentation pathways of Br-ketamine.

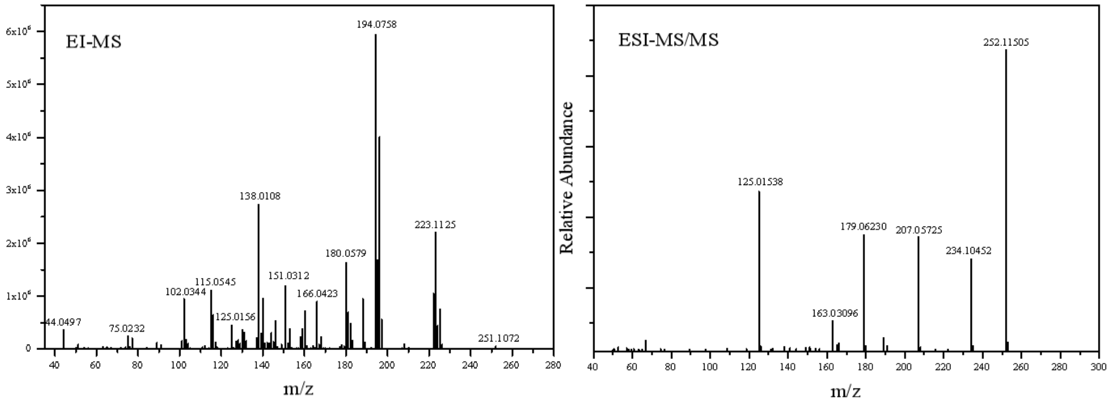

Scheme S11. The EI-MS and ESI-MS/MS spectra of NENK.

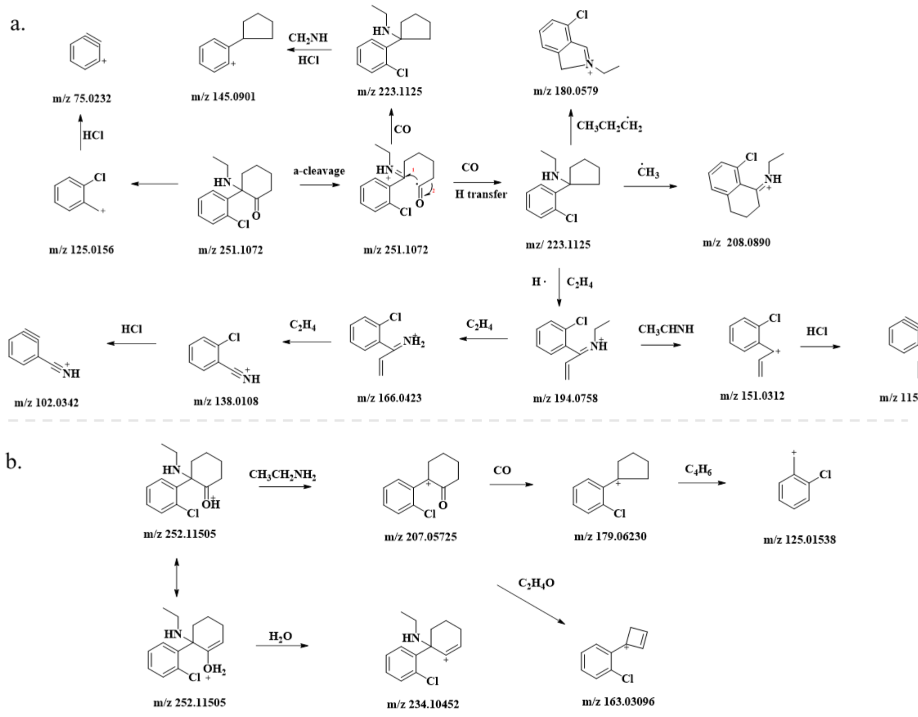

Scheme S12. a). The EI-MS and b). ESI-MS/MS fragmentation pathways of NENK.

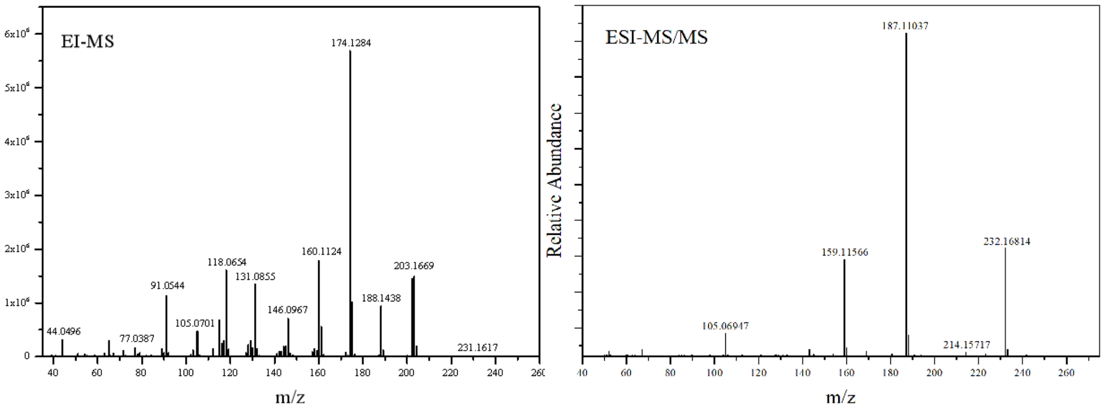

Scheme S13. The EI-MS and ESI-MS/MS spectra of DMXE.

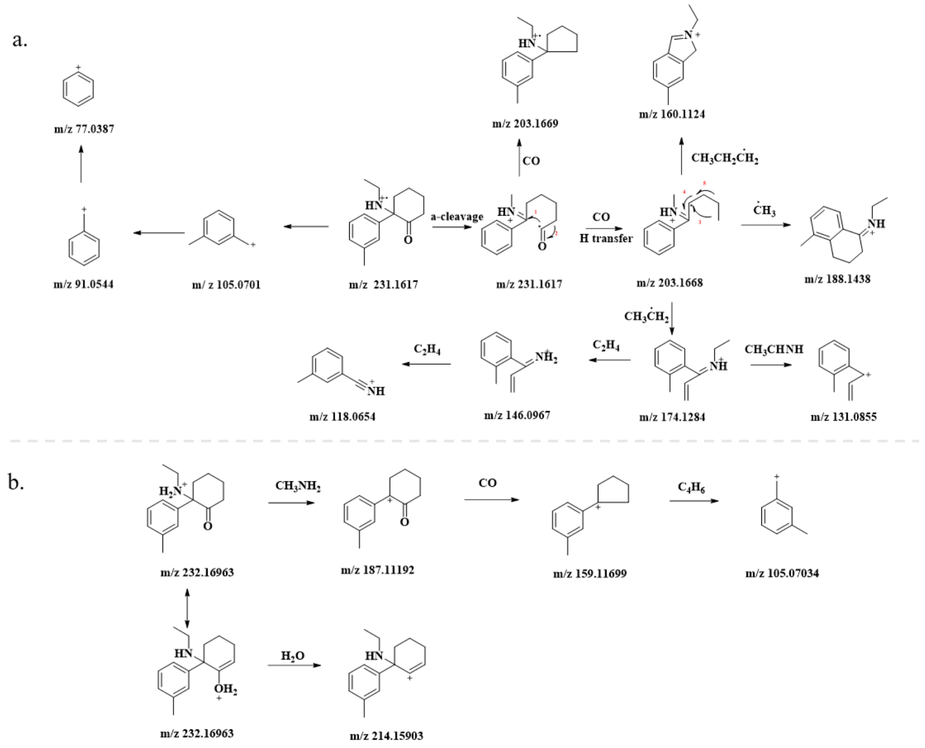

Scheme S14. a). The EI-MS and b). ESI-MS/MS fragmentation pathways of DMXE.

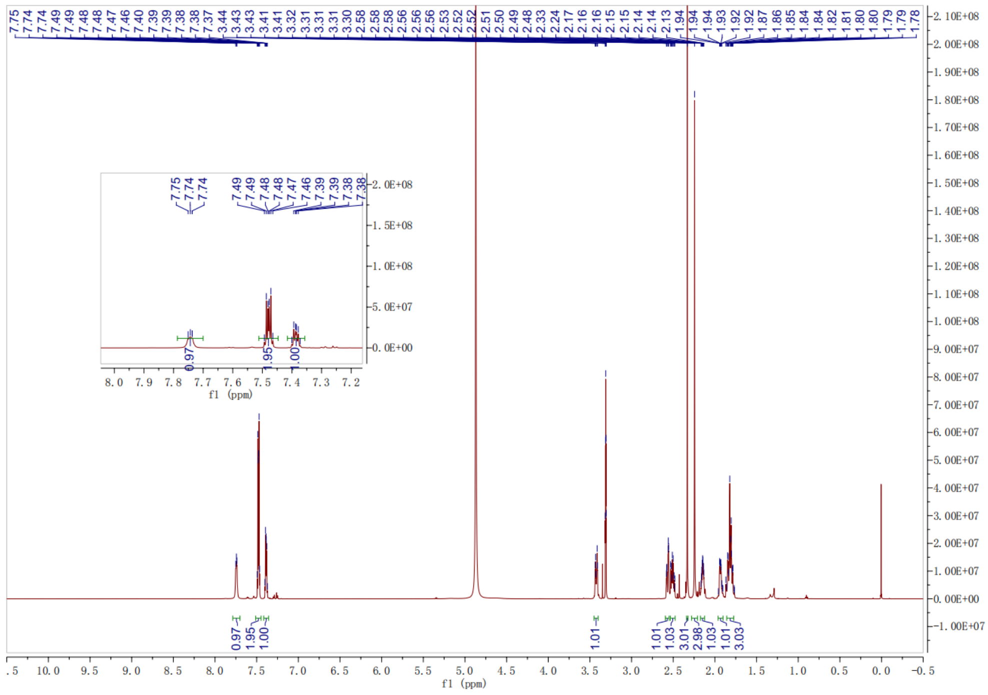

Scheme S15. The ^{1}H NMR spectra of compound 1.

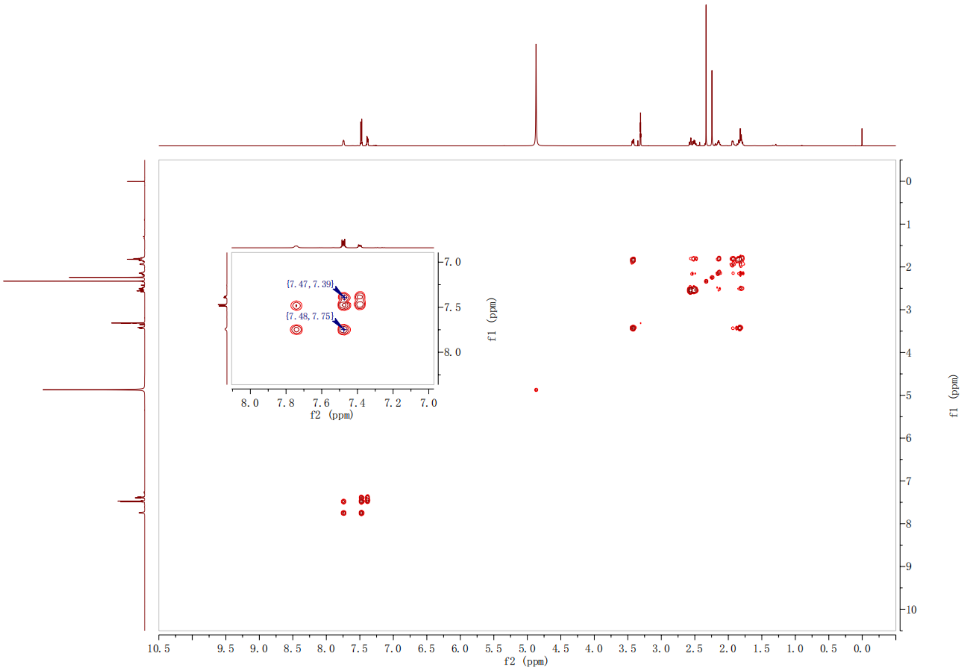

Scheme S16. The $^{1}H/^{1}H$ COSY spectra of compound 1.
